# NINJ1-mediated membrane rupture bridges cell death to tissue inflammation

**DOI:** 10.1042/BST20260842

**Published:** 2026-10-13

**Authors:** Thu Uyen Tran, Quan Thinh Le, Tuong-Khanh My Tu, Thanh Kha Phan

**Affiliations:** Department of Biochemistry and Chemistry, Research Centre for Extracellular Vesicles, La Trobe Institute for Molecular Science, La Trobe University, Melbourne, VIC 3086, Australia

**Keywords:** cell death, inflammation, membrane rupture, NINJ1, ninjurin-1

## Abstract

Plasma membrane rupture (PMR) is a terminal event in many forms of lytic cell death, yet was long regarded as a passive consequence of osmotic swelling and mechanical membrane failure. The identification of ninjurin-1 (NINJ1) as a key mediator of PMR fundamentally changed this view by establishing that membrane permeabilisation and terminal membrane rupture are mechanistically distinct processes. Across different cell death modalities, NINJ1 promotes the transition from membrane damage to catastrophic cellular disintegration. Structural studies have revealed how NINJ1 is restrained by autoinhibition in healthy cells and assembles into higher-order membrane-remodelling complexes following activation. However, how NINJ1 is activated and how its assemblies cooperate with the physical state of the dying cell to produce membrane rupture remain incompletely understood. NINJ1-dependent PMR also determines the release of intracellular contents, linking cell death to inflammation, host defence, tissue homeostasis and disease. Emerging evidence suggests that NINJ1 integrates molecular, metabolic and biophysical signals and can function beyond canonical cell-death pathways. Here, we discuss the molecular mechanisms governing NINJ1-mediated PMR, how diverse cell death pathways converge on this process, and how terminal membrane rupture shapes inflammatory responses, host defence and tissue pathology. We further consider unresolved mechanisms of NINJ1 activation and the therapeutic potential of uncoupling cell death from inflammatory membrane rupture.

## Introduction

Cell death is fundamental to development, tissue homeostasis and host defence, but its biological consequences extend beyond the activation of cell death pathways themselves. Following loss of viability, the fate of dying cells is determined in part by the integrity of the plasma membrane, which governs whether intracellular contents remain contained for controlled clearance or are released into the extracellular environment. For clarity, plasma membrane permeabilisation is used herein to denote an increase in membrane permeability that permits passage of ions, small molecules or fluorescent dyes across the plasma membrane, whereas plasma membrane rupture (PMR) refers to more extensive loss of membrane barrier function that permits release of large intracellular components such as LDH, HMGB1 and other damage-associated molecular patterns (DAMPs). These events can be temporally and mechanistically separable: permeabilisation can occur without immediate PMR, and morphological collapse or ‘bursting’ is neither required nor sufficient to define rupture. Experimentally, membrane permeabilisation is commonly assessed by uptake of membrane-impermeant dyes, whereas PMR is commonly assessed by release of macromolecular intracellular constituents such as LDH or HMGB1, often together with live-cell imaging and morphological analysis. The timing and extent of PMR therefore critically influence the inflammatory output of cell death by determining when and to what extent intracellular DAMPs and other cellular contents become exposed to the extracellular environment [1,2]. PMR is therefore not merely the terminal stage of cellular demise, but a critical interface linking cell death to tissue inflammation.

For decades, PMR was widely regarded as a passive consequence of osmotic swelling and mechanical membrane failure during lytic cell death. Recent evidence, however, shows that cells can lose viability and become membrane-permeable without undergoing full catastrophic rupture. The discovery of ninjurin-1 (NINJ1) as a key mediator of PMR provided a molecular basis for this phenomenon, demonstrating that cell death execution can be uncoupled from terminal cellular rupture [3,4]. NINJ1-dependent PMR has since been implicated downstream of multiple forms of regulated cell death, suggesting that diverse death pathways converge on a common rupture programme that governs the transition from intracellular death signalling to extracellular inflammatory responses [3,5].

NINJ1 is a transmembrane protein that undergoes regulated oligomerisation to form higher-order membrane-associated assemblies capable of remodelling and disrupting lipid bilayers [4]. Recent structural studies have begun to define the molecular basis of this activity, revealing both the architecture of rupture-competent NINJ1 assemblies and an autoinhibited conformation that constrains oligomerisation in viable cells [4,6,7]. Despite these advances, fundamental questions remain unresolved. It is unclear how signals generated by upstream cell death pathways initiate NINJ1 activation, how NINJ1 activity is coordinated with changes in membrane mechanics, cytoskeletal integrity and cellular swelling, and whether NINJ1 functions as an autonomous membrane-disrupting machine or instead acts by amplifying pre-existing membrane damage to drive catastrophic rupture [7–9].

In this mini-review, we discuss the molecular mechanisms that govern NINJ1-mediated PMR, examine how diverse cell death pathways converge on this terminal event, and explore the consequences of membrane rupture for DAMP release, inflammation and tissue homeostasis. We further highlight emerging questions surrounding NINJ1 activation and regulation, and consider the therapeutic potential of uncoupling cell death from inflammatory lysis.

## Mechanisms of NINJ1-mediated membrane rupture in cell death

### From inactive NINJ1 to membrane-rupturing assemblies

The ability of NINJ1 to drive PMR depends on its transition from an inactive membrane-resident protein to a higher-order oligomeric assembly. Initial studies identified NINJ1 as a critical mediator of PMR across multiple forms of lytic cell death and showed that oligomerisation is required for this activity [3,4]. Notably, NINJ1-deficient cells still undergo cell death but fail to progress efficiently to terminal membrane rupture, instead developing persistent membrane blebs and balloon-like structures. These observations established that NINJ1 acts downstream of cell death execution to mediate terminal plasma membrane disintegration [3].

Structural studies revealed that NINJ1 subunits polymerise into extended filamentous structures through interactions involving transmembrane and amphipathic helices. NINJ1 also forms large filamentous and ring-like assemblies in dying cells, linking higher-order oligomerisation to its membrane-disrupting activity [4,6]. NINJ1 filaments are assembled through intermolecular interactions involving amphipathic helices, with exposed hydrophilic faces mediating interactions between neighbouring subunits. In the resting state, these hydrophilic faces are buried within a face-to-face NINJ1 dimer, restraining productive higher-order assembly. Destabilisation of this dimer induces a kink in transmembrane helix 1 and favours transition to a polymerisation-competent conformation, whereas mutations that stabilise the dimer suppress NINJ1-mediated PMR [7]. Notably, higher-order NINJ1 complexes can also be detected in healthy cells by native gel electrophoresis, suggesting that NINJ1 assembly is not synonymous with membrane rupture and that additional regulatory steps control progression to a rupture-competent state [3].

How this switch is engaged during cell death remains poorly understood. Cell swelling promotes NINJ1 oligomerisation in several forms of regulated necrosis, suggesting that the biophysical consequences of cell death contribute to activation. However, whether NINJ1 is triggered by specific upstream signals, progressive changes in membrane architecture, or both remains unresolved [9].

### How does NINJ1 rupture plasma membrane?

Although NINJ1 assembly is required for PMR, how these assemblies ultimately disrupt the plasma membrane remains incompletely understood. Unlike lytic cell death-associated pore-forming effector proteins (e.g. gasdermins and MLKL), NINJ1 oligomers do not appear to function primarily by forming conventional transmembrane pores [4,6,10]. Instead, two structural models have been proposed. Degen et al. showed that NINJ1 forms filamentous assemblies that associate with membrane edges and proposed that these structures stabilise membrane lesions, potentially through a zipper-like mechanism [4]. By contrast, David et al. identified ring-like NINJ1 assemblies that encircle and excise membrane discs in a “cookie-cutter” mechanism [6]. These models are not necessarily mutually exclusive and may represent distinct structural states or mechanisms of NINJ1-mediated membrane disruption.

The distinction between membrane permeabilisation and terminal PMR is particularly evident during pyroptosis. Gasdermin D (GSDMD) pores induce extensive membrane permeabilisation and loss of viability, yet pyroptotic cells can remain membrane-confined despite profound permeability defects. NINJ1 promotes progression from this permeabilised state to terminal rupture, demonstrating that membrane permeabilisation and PMR are mechanistically distinct events [3]. Quantitative analyses of pyroptotic cell-volume dynamics further reveal an early GSDMD-dependent swelling phase followed by a later NINJ1-dependent phase associated with formation of larger membrane lesions [11].

The physical state of the dying cell further determines whether NINJ1-dependent lesions progress to PMR. Increased NINJ1 abundance lowers the force required for mechanically induced rupture, whereas NINJ1 deficiency increases resistance to membrane rupture under mechanical strain [8]. Recent work supports a two-step mechanism in which NINJ1 first assembles into membrane-embedded filaments, followed by swelling-induced increases in membrane tension that promote opening and propagation of these lesions into PMR. Preventing cell swelling inhibits PMR without disrupting NINJ1 oligomerisation, indicating that NINJ1 assembly and lesion opening are separable events [12].

NINJ1 therefore operates at the interface between molecular membrane damage and cellular mechanics. NINJ1 assembly generates rupture-competent membrane lesions, while the physical state of the dying cell determines whether these lesions open and propagate into catastrophic PMR. Whether the relative contribution of these mechanisms differs across cell-death modalities and tissue environments remains an important unresolved question.

### Convergence of cell death pathways on NINJ1-dependent rupture

A major conceptual advance arising from the discovery of NINJ1 is the recognition that membrane permeabilisation and PMR are distinct biological processes. Multiple cell death pathways employ dedicated effectors to compromise membrane integrity, including gasdermins during pyroptosis and MLKL during necroptosis [13]. These effectors disrupt membrane barrier function, promoting ionic flux, osmotic imbalance and loss of viability. However, membrane permeabilisation alone does not necessarily result in catastrophic rupture or large-scale release of intracellular contents. Instead, NINJ1 can provide a downstream membrane-disrupting mechanism that converts membrane damage into terminal cellular disintegration across several forms of lytic cell death (Figure 1) [3,5,9].

**Figure 1:**
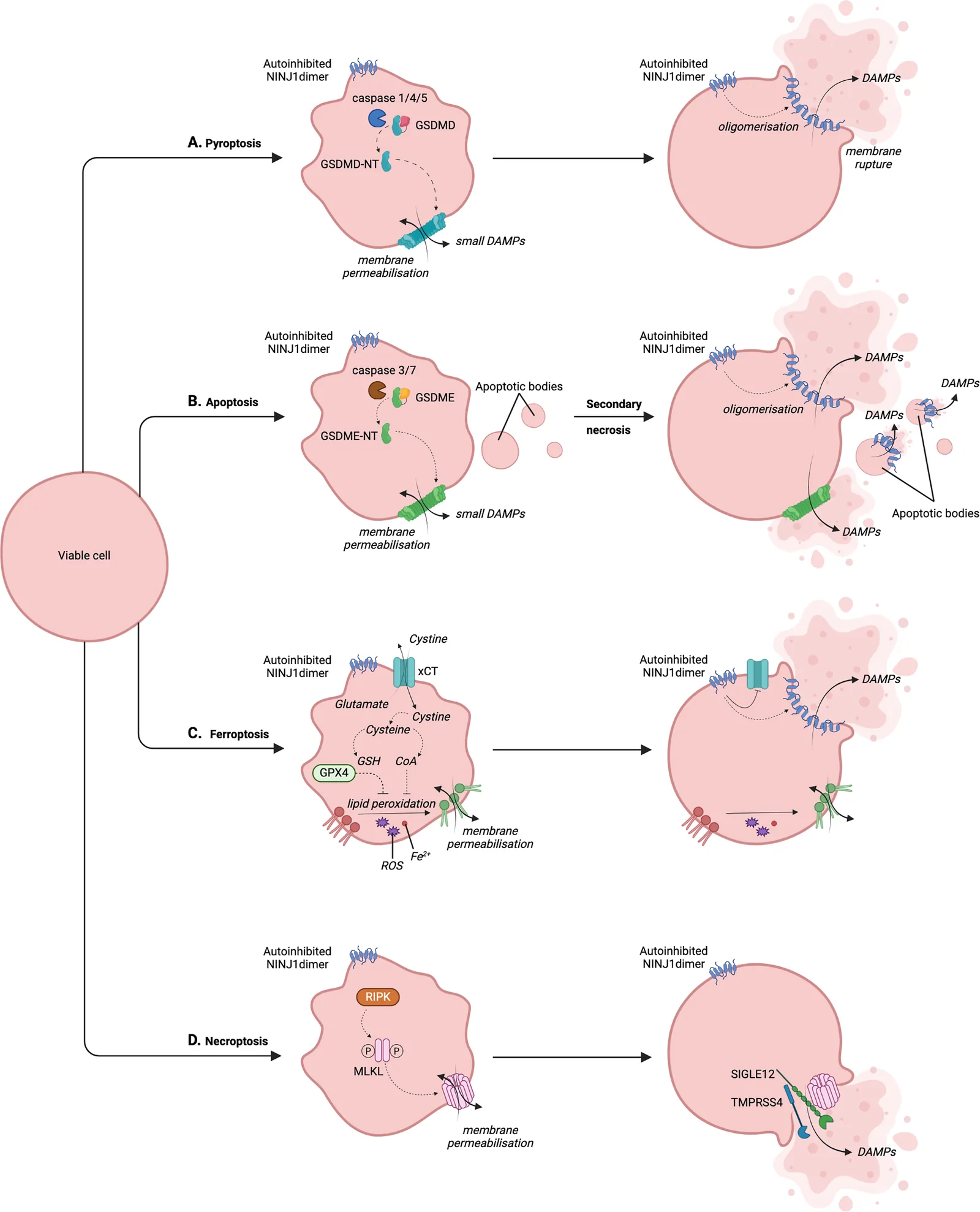
NINJ1 mediates PMR in different cell death pathways (**A**) During pyroptosis, inflammatory caspases -1, -4, and -5 cleave (GSDMD) to liberate an active N-terminal fragment (GSDMD-NT). Upon translocation to plasma membrane, GSDMD-NT monomers bind phospholipids and form oligomeric transmembrane pores that cause intial membrane permeabilisation. Subsequently, NINJ1 is activated and oligomerises to promote terminal membrane rupture, leading to large-scale release of intracellular contents. (**B**) Following apoptosis, GSMDE cleavage by apoptotic caspase-3/7 generates a pore-forming N-terminal fragment that can promote membrane permeabilisation, which may proceed to lytic second necrosis in certain settings. In GSDME-independent secondary necrosis, NINJ1 oligomerisation occurs after apoptotic body biogenesis (also known as ‘apoptotic cell disassembly’) to promote terminal membrane rupture. (**C**) During ferroptosis, lipid peroxidation triggered by ROS and iron catalysis cause initial plasma membrane permeabilisation and ionic imbalance. Subsequently, NINJ1 promotes PMR and DAMP release. NINJ1 further exacerbates PMR through inhibition of xCT transporter, which imports cystine for glutathione and coenzyme A metabolism that inhibits ferroptosis. (**D**) PMR in necroptosis occurs after MLKL phosphorylation and pore formation and can be independent of NINJ1, but instead mediated by TMPRSS2 cleavage of SIGLEC12. NINJ1: ninjurin-1, GSDMD: gasdermin D, GSDMD-NT: GSDMD N-terminal fragment, DAMPs: danger-associated molecular patterns, GSDME: gasdermin E, GSDME-NT: GSDME N-terminal fragment, MLKL: mixed lineage kinase domain-like pseudokinase, xCT: cystine/glutamate antiporter, GSH: glutathione, CoA: coenzyme A, GPX4: glutathione peroxidase 4, ROS: reactive oxygen species, SIGLEC12: sialic acid-binding Ig-like lectin 12, TMPRSS4: transmembrane protease serine 4. This figure was created using BioRender (https://www.biorender.com).

During pyroptosis, inflammatory caspases (caspases-1, -4, and -5 in humans) cleave GSDMD to generate an active N-terminal fragment, GSDMD-NT, that acts as the principal membrane-disrupting effector of pyroptotic cell death. Upon activation, GSDMD-NT monomers bind phospholipids and oligomerise to form transmembrane pores that compromise plasma membrane integrity. These pores promote ionic flux, osmotic imbalance and cell swelling, but do not necessarily cause immediate terminal PMR [14]. NINJ1 instead promotes the subsequent membrane breakdown required for large-scale release of intracellular contents [3]. While NINJ1-deficient cells undergo GSDMD cleavage and pore formation, they fail to complete terminal rupture. This functional separation demonstrates that membrane permeabilisation and PMR are distinct, sequential processes driven by different molecular mechanisms [3].

Secondary necrosis was once considered an unregulated outcome of apoptosis upon defective dead cell clearance (or ‘efferocytosis’). It is now recognised as a regulated post-apoptotic process involving progressive loss of plasma membrane integrity, which can encompass membrane permeabilisation and, ultimately, terminal PMR. Importantly, the experimental definition of secondary necrosis varies with the assay used: uptake of membrane-impermeant dyes such as SYTOX or 7-AAD primarily reports increased membrane permeability and does not by itself establish catastrophic PMR. Recent studies have implicated both gasdermin E (GSDME) and NINJ1 in this transition. Like GSDMD, GSDME cleavage by apoptotic caspase-3/7 generates a pore-forming N-terminal fragment that can promote membrane permeabilisation following apoptosis [15,16], whereas NINJ1 oligomerisation occurs later, following the completion of structural breakdown of an apoptotic cell (also known as ‘apoptotic cell disassembly’), to promote terminal PMR [9,17]. However, the requirement for GSDME appears to be context dependent. De Schutter et al. showed that GSDME deficiency impaired influx of fluorescent dextrans during apoptosis-driven secondary necrosis but did not affect their efflux, suggesting that GSDME contributes to membrane permeabilisation but may not be essential for all aspects of post-apoptotic intracellular-content release [16]. Similarly, Gavali et al. found that GSDME deficiency does not compromise the kinetics of secondary necrosis in diphtheria toxin or cisplatin-induced apoptosis despite prominent GSDME cleavage, as assessed by SYTOX or 7-AAD [18]. It is possible that GSDME and NINJ1 make distinct and context-dependent contributions to the transition from apoptosis to secondary necrosis rather than functioning within a simple linear cascade. Moreover, recent work from our group identified a calcium influx-dependent, Atg9A-mediated mechanism to delay secondary necrosis, without altering NINJ1 or GSDME oligomerisation, suggesting that membrane repair provides an additional checkpoint between apoptotic membrane damage and terminal PMR [19]. Together, these findings suggest that the onset of secondary necrosis is actively regulated rather than representing an inevitable consequence of apoptotic cell death. From the perspective of tissue homeostasis, this regulation is necessary to allow sufficient time for efficient dead cell clearance and limit premature exposure of intracellular inflammatory contents.

Ferroptosis, triggered by iron-catalysed lipid peroxidation as well as a failure of glutathione- and coenzyme A-mediated antioxidant defences, further illustrates the context dependence of NINJ1 function. Phospholipid peroxides cause initial plasma membrane permeabilisation and ionic imbalance. Subsequently, NINJ1 promotes PMR and DAMP release. Moreover, NINJ1 also regulates ferroptotic sensitivity through a distinct interaction with the xCT transporter, which imports cystine for glutathione and coenzyme A metabolism [5,21]. These findings separate the membrane-disrupting and metabolic functions of NINJ1 and raise the possibility that its contribution to cell death extends beyond terminal PMR.

The relationship between NINJ1 and necroptosis is more complex. In human cells, PMR downstream of MLKL can proceed independently of NINJ1 and may instead be mediated by SIGLEC12, which undergoes proteolytic processing and assembles into fibrillar structures. By contrast, this requirement for SIGLEC12 was not observed in mouse cells, suggesting that this mechanism may be species specific [20]. Thus, NINJ1 should not be viewed as a universal terminal effector downstream of all lytic death pathways.

Collectively, these findings support a model in which distinct death pathways generate membrane damage through pathway-specific mechanisms, while NINJ1 functions as a major but context-dependent mediator of the transition from membrane damage to terminal rupture. Rather than representing a universal endpoint downstream of every lytic death programme, NINJ1 defines one important route by which dying cells convert loss of membrane integrity into inflammatory cellular disintegration.

### What activates NINJ1?

Despite recent structural advances, how NINJ1 is activated during cell death remains incompletely understood. Structural studies have established that NINJ1 is maintained in an autoinhibited dimeric state in healthy cells and must undergo a conformational transition before forming higher-order assemblies capable of disrupting the plasma membrane. However, the signals that release this autoinhibition remain unclear [7].

One possibility is that NINJ1 is activated by specific molecular signals generated downstream of cell death pathways. Yet, despite NINJ1-dependent PMR occurring across pyroptosis, secondary necrosis and ferroptosis, no common biochemical activator has emerged. Indeed, reactive oxygen species are neither sufficient nor required for NINJ1-dependent PMR in several regulated-necrosis settings. By contrast, calcium influx has emerged as a potential upstream signal. Calcium entry may promote NINJ1 clustering through the calcium-activated phospholipid scramblase TMEM16F, with calcium-dependent lipid scrambling required for NINJ1 clustering and rupture in macrophages [22]. This mechanism also operates downstream of P2X7 receptor activation and does not require inflammasomes, pannexins or GSDMD, suggesting that changes in plasma membrane lipid organisation can directly engage NINJ1. However, NINJ1 activation may not be governed by calcium alone. Hypotonic swelling can induce NINJ1 oligomerisation in several forms of regulated necrosis, suggesting that the biophysical consequences of cellular demise contribute to activation [9]. Importantly, higher-order NINJ1 assembly does not necessarily equate to terminal PMR. Filamentous NINJ1 assemblies can form under conditions where membrane rupture is prevented, indicating that oligomerisation and lesion formation can be experimentally separated from lesion opening and catastrophic membrane failure [9,12]. Membrane architecture, tension and cellular mechanics may cooperate with conformational activation to determine whether assembled NINJ1 becomes rupture competent [9,23].

Recent findings further challenge a model in which NINJ1 is exclusively downstream of canonical cell-death pathways. Infection-induced glucose starvation can trigger NINJ1-dependent macrophage lysis independently of known cell-death programmes, while alanine suppresses this response by inhibiting NINJ1 oligomerisation [24]. Conversely, TRIM72-mediated ubiquitination of NINJ1 promotes its degradation and restrains PMR, demonstrating that NINJ1 activity is also controlled at the level of protein abundance [25].

Together, these findings suggest that NINJ1 activation is unlikely to be governed by a single upstream death signal. Rather, NINJ1 may integrate molecular, metabolic and biophysical cues to determine when membrane-rupturing assemblies form and whether these assemblies progress to catastrophic PMR. In this view, NINJ1 functions not merely as a downstream executor of cell death but as an integration point that couples cellular stress to terminal membrane rupture. Defining how these signals converge on NINJ1 will be central to understanding how PMR is controlled across diverse cellular contexts.

## Consequences of NINJ1-mediated membrane rupture

### DAMP release and inflammation

PMR fundamentally alters the extracellular consequences of cell death by enabling the release of intracellular contents that remain largely contained during earlier stages of membrane damage. NINJ1-deficient cells undergo cell death yet remain in a ballooned state and exhibit markedly reduced release of intracellular proteins, including HMGB1 and LDH, demonstrating that terminal PMR is required for efficient release of macromolecular cellular contents. NINJ1 therefore does not simply determine whether a dying cell loses membrane integrity; it determines whether membrane damage progresses to terminal rupture and broad exposure of intracellular material to the extracellular environment [3]. This distinction is also evident during ferroptosis, where NINJ1 is dispensable for lipid peroxidation, calcium influx and cell swelling but is required for subsequent PMR and DAMP release [5].

By separating cell death from terminal cellular disintegration, NINJ1 functions as a checkpoint linking death execution to inflammatory signalling. NINJ1-dependent PMR enables the extracellular exposure of intracellular danger signals, including HMGB1 and other DAMPs, providing a mechanism through which cellular disintegration can propagate inflammatory responses beyond the dying cell [3].

Collectively, these findings identify NINJ1-dependent PMR as a critical determinant of the inflammatory consequences of cell death. By controlling the release of intracellular danger signals, NINJ1 helps determine the transition from cell-autonomous death to tissue-level inflammation.

### Host defence

Lytic cell death can promote host defence by eliminating infected cells and exposing intracellular contents to the immune system. NINJ1-dependent PMR extends these effects beyond the dying cell by enabling the release of intracellular danger signals following terminal membrane rupture. In this context, NINJ1 can couple cellular disruption to recruitment and activation of surrounding immune cells, reinforcing inflammatory responses that contribute to pathogen control.

This role is evident during bacterial infection, where impaired NINJ1 expression or cell lysis increases susceptibility to *Yersinia* despite preserved upstream inflammatory caspase activation and GSDMD cleavage. These findings indicate that terminal rupture contributes to host defence beyond upstream pyroptotic execution [26]. NINJ1 can also be directly co-opted by pathogens. Murine norovirus recruits NINJ1 to viral replication complexes to facilitate NS1 secretion, while NINJ1-dependent PMR also promotes extracellular release of viral particles and host-derived factors [17,27]. More recently, infection-induced glucose starvation was shown to trigger NINJ1-dependent macrophage rupture during *Candida* infection independently of canonical cell-death pathways, promoting fungal egress from macrophages [24]. Together, these findings reveal that NINJ1-mediated PMR can both reinforce antimicrobial responses and be exploited by pathogens to escape cellular containment.

Thus, the consequences of NINJ1-dependent PMR during infection are context dependent. Rupture can amplify inflammatory responses that support antimicrobial defence, but excessive or pathogen-exploited rupture can promote tissue injury or pathogen dissemination. NINJ1 therefore sits at a critical intersection between cellular containment and tissue-level immune activation, with its consequences determined by the infectious and tissue context.

### Apoptotic cell disassembly, efferocytosis, and tissue homeostasis

Apoptotic cells undergo regulated disassembly into membrane-bound apoptotic bodies, which facilitates their recognition and clearance by phagocytes through efferocytosis [28,29]. Efficient efferocytosis normally removes apoptotic cells before loss of membrane integrity and secondary necrosis, thereby limiting extracellular exposure to intracellular DAMPs and maintaining inflammatory homeostasis. When clearance is overwhelmed or defective, apoptotic cells can progress to secondary necrosis and undergo PMR, converting a normally non-inflammatory death process into one capable of promoting inflammation.

NINJ1 appears to act at this transition rather than during apoptotic cell disassembly itself. NINJ1-deficient cells undergo normal apoptotic cell disassembly and generate apoptotic bodies, indicating that NINJ1 is not required for this process [17]. Instead, we demonstrated that NINJ1 oligomerisation increases after apoptotic cell disassembly, consistent with a later role in regulating the stability and subsequent rupture of membrane-bound apoptotic corpses [17]. Indeed, NINJ1 oligomerises on these apoptotic bodies and promotes their membrane rupture and cellular content release, extending its role beyond the intact dying cell to post-apoptotic extracellular structures.

These findings suggest that NINJ1 may determine the balance between corpse containment and extracellular disintegration after apoptosis. By delaying the loss of membrane integrity, efferocytosis can then maintain the immunologically silent outcome of cell death and promote tissue repair, whereas NINJ1-mediated PMR may expose intracellular contents when apoptotic remnants persist or escape clearance. Whether NINJ1-dependent rupture directly alters efferocytic recognition, phagocyte responses or subsequent tissue homeostasis remains poorly understood. Defining how NINJ1 intersects with the temporal transition from apoptotic cell disassembly to secondary necrosis may therefore reveal an important mechanism linking the physical fate of dying-cell remnants to inflammation resolution and tissue homeostasis.

### NINJ1 in diseases and therapeutic opportunities

NINJ1-dependent PMR can amplify tissue injury by converting cell death into extracellular DAMP release and sustained inflammatory signalling. Particularly, in sterile tissue injury, excessive lytic cell death can propagate inflammation beyond the initiating insult. In mouse models of hepatocellular injury, an anti-NINJ1 monoclonal antibody blocked NINJ1 oligomerisation, reduced PMR and HMGB1 and IL-18 release, and attenuated neutrophil infiltration and tissue injury, including following hepatic ischaemia reperfusion [30]. Similarly, myeloid NINJ1 deletion reduced HMGB1 release and NET formation *in vivo*, thus mitigating oxalate nephropathy, whilst NINJ1 antibody treatment reduced DAMP release and neuroinflammation while improving functional recovery [31,32].

More recent work suggests that NINJ1 can also contribute to persistent tissue injury by linking PMR to sustained inflammatory remodelling. In AKI-to-CKD progression, TRIM72-mediated ubiquitination limits NINJ1 abundance and PMR, whereas loss of TRIM72 stabilises NINJ1, enhances HMGB1 release and promotes inflammatory and fibrotic responses [25]. NINJ1-dependent PMR can also contribute to inflammasome-associated coagulation and inflammation [33]. Together, these findings suggest that NINJ1 may contribute not only to acute tissue injury but also to persistent pathology driven by unresolved cellular disintegration.

The therapeutic appeal of NINJ1 lies in its position downstream of cell-death execution. Targeting NINJ1 could therefore suppress inflammatory PMR and DAMP release without broadly inhibiting the upstream death pathways required for tissue defence and homeostasis. However, the contribution of NINJ1-dependent PMR to host defence and its influence on pathogen containment may raise the possibility that systemic inhibition could impair beneficial responses to infection. The effects of NINJ1 blockade on efferocytosis, tissue repair and cell-type-specific host defence also remain poorly defined. Thus, the therapeutic opportunity may ultimately depend on context-selective inhibition of NINJ1-mediated PMR rather than indiscriminate suppression of cell lysis.

## Concluding remarks

The emergence of NINJ1 as a regulator of PMR has fundamentally shifted our understanding of the terminal stages of cell death. A growing body of evidence supports a model in which cell-death stimuli engage distinct execution pathways that cause loss of viability and membrane damage, followed by NINJ1 activation, membrane rupture and release of intracellular contents that shape the response of the surrounding tissue. In this framework, NINJ1 does not necessarily determine whether a cell dies, but it instead helps determine how the dead cell physically disintegrates and communicates its demise to the surrounding tissue. Several fundamental questions remain unresolved, including how NINJ1 is activated in response to distinct forms of cellular stress, what drives productive oligomerisation, how NINJ1 assemblies cooperate with membrane tension, cell swelling and other biomechanical forces to execute rupture, and whether NINJ1 functions primarily as an autonomous membrane-disrupting machinery or as a context-dependent amplifier of membrane damage. Resolving these questions will define how terminal PMR is controlled and clarify how the physical fate of dying cells shapes inflammation, host defence and tissue pathology.

## Perspectives

- PMR is a critical determinant of how dying cells communicate with their surroundings, yet its regulation has only recently emerged as a distinct biological process. Understanding this transition is therefore essential for explaining how cell death is translated into inflammation and tissue injury.
- NINJ1 has emerged as a major mediator of terminal membrane rupture that acts downstream of diverse cell-death programmes, converting membrane damage into catastrophic cellular disintegration and intracellular content release. Current evidence suggests that NINJ1 assembly is controlled by molecular, metabolic and biophysical cues, while its contribution to rupture varies across cell-death and tissue contexts.
- Key priorities are to define how NINJ1 escapes autoinhibition, how its assemblies interact with membrane mechanics to execute rupture, and how these mechanisms differ across cell types and death modalities. Resolving these questions may enable context-selective targeting of NINJ1 to limit pathological inflammation while preserving beneficial cell-death and host-defence responses.

## Open Access

Open access for this article was enabled by the participation of La Trobe University in an all-inclusive Read & Publish agreement with Portland Press and the Biochemical Society under an agreement with CAUL.

## Abbreviations

CoA: coenzyme A
DAMPs: damage-associated molecular patterns
GSDMD: Gasdermin D
GSDMD-NT: GSDMD N-terminal fragment
GSDME: gasdermin E
GSDME-NT: GSDME N-terminal fragment
GSH: glutathione
GPX4: glutathione peroxidase 4
MLKL: mixed lineage kinase domain-like pseudokinase
NINJ1: ninjurin-1
PMR: Plasma membrane rupture
ROS: reactive oxygen species
SIGLEC12: sialic acid-binding Ig-like lectin 12
TMPRSS4: transmembrane protease serine 4
xCT: cystine/glutamate antiporter
